# Glycosyltransferase FvCpsA Regulates Fumonisin Biosynthesis and Virulence in *Fusarium verticillioides*

**DOI:** 10.3390/toxins13100718

**Published:** 2021-10-11

**Authors:** Qi Deng, Hanxiang Wu, Qin Gu, Guangfei Tang, Wende Liu

**Affiliations:** 1 State Key Laboratory for Biology of Plant Diseases and Insect Pests, Institute of Plant Protection, Chinese Academy of Agricultural Sciences, Beijing 100193, China; qidengDQ@163.com (Q.D.); wuhanxiang@caas.cn (H.W.); 2 Department of Plant Pathology, College of Plant Protection, Nanjing Agricultural University, Key Laboratory of Monitoring and Management of Crop Diseases and Pest Insects, Ministry of Education, Nanjing 210095, China; guqin@njau.edu.cn

**Keywords:** *Fusarium verticillioides*, family 2 glycosyltransferase (GT2), fumonisin B1, virulence

## Abstract

*Fusarium verticillioides* is the major maize pathogen associated with ear rot and stalk rot worldwide. Fumonisin B1 (FB1) produced by *F. verticillioides*, poses a serious threat to human and animal health. However, our understanding of FB1 synthesis and virulence mechanism in this fungus is still very limited. Glycosylation catalyzed by glycosyltransferases (GTs) has been identified as contributing to fungal infection and secondary metabolism synthesis. In this study, a family 2 glycosyltransferase, FvCpsA, was identified and characterized in *F. verticillioides*. Δ*FvcpsA* exhibited significant defects in vegetative growth. Moreover, Δ*FvcpsA* also increased resistance to osmotic and cell wall stress agents. In addition, expression levels of *FUM* genes involved in FB1 production were greatly up-regulated in Δ*FvcpsA*. HPLC (high performance liquid chromatography) analysis revealed that Δ*FvcpsA* significantly increased FB1 production. Interestingly, we found that the deletion of *FvCPSA* showed penetration defects on cellophane membrane, and thus led to obvious defects in pathogenicity. Characterization of FvCpsA domain experiments showed that conserved DXD and QXXRW domains were vital for the biological functions of FvCpsA. Taken together, our results indicate that FvCpsA is critical for fungal growth, FB1 biosynthesis and virulence in *F. verticillioides*.

## 1. Introduction

The filamentous ascomycete *Fusarium verticillioides* is one of the most important fungal pathogens causing stalk and ear rot on maize [1]. *F. verticillioides* results in substantial maize yield losses. Moreover, various types of mycotoxins, such as fumonisins, fusarins and fusaric acid produced by *F. verticillioides*, lead to severe human and animal diseases [2]. Fumonisins are the most notorious mycotoxins, which have been widely distributed among corn and corn-based foods [3,4]. Among the diverse forms of the fumonisins, fumonisin B1 (FB1) is one of the most common fumonisins [5]. The biosynthetic gene cluster for FB1, including 17 *FUM* genes, has been identified and described [6]. FB1 synthesis appears to be regulated by various environmental factors, such as pH and nutrient sources, at the transcriptional level [7]. The mitogen-activated protein kinase (MAPK) signaling pathways have also been shown to regulate FB1 production in *F. verticillioides* [8]. Recent studies have shown that post-translational modifications, such as acetylation, methylation and phosphorylation, play a critical role in FB1 production [9]. To date, the molecular mechanisms underlying the regulation of FB1 are still unclear.

Glycosylation is a major post-translational modification of proteins and serves in many biological processes by modulating the folding, stability and function of proteins [10]. Glycosyltransferases (GTs) are the enzymes that catalyze glycosylation. These enzymes are distributed in both prokaryotes and eukaryotes and transfer sugar residue from an activated nucleotide sugar donor onto saccharide or non-saccharide receivers, formulating glycosidic bonds for glycosylation process [11]. Based on crystal structure, GTs are classified into three types: GT-A, GT-B and GT-C families. Based on sequence, GTs have been classified into a number of sequence similarity-based families in the Carbohydrate-Active Enzymes (CAZy) database in 1998 (http://www.cazy.org, accessed on 1 January 2015). Among them, Glycosyltransferase family 2 (GT2) is one of the largest families [12,13,14]. Members of GT2 are involved not only in biomass synthesis, but also in many of the intricate details of cellular processes [12]. Great advances in the exploration of biological function in the GT superfamily have been made over the past few decades in plant and plant pathogenic fungi [15]. However, the function of individual enzymes in filamentous fungi remains to be fully elucidated.

A growing collection of evidence shows that glycosylation plays an important function in fungal infection and host immune response in the early stages of infection [16]. In *Zymoseptoria tritici*, the gene *ZtGT2,* which is predicted to encode a family 2 glycosyltransferase, regulates hyphal growth, and is essential for virulence in wheat. The deletion mutant of the *ZtGT2* homologous gene in the fungus *Fusarium graminearum*, Δ*FgGT2*, also shows significantly impaired hyphal growth and virulence in wheat. It is worth mentioning that *GT2* homologous genes are widely distributed in most ascomycete filamentous fungi but are absent in yeast species that do not form true hyphae [17]. *Magnaporthe oryzae, MoGT2*, also exhibits a critical role in hyphal growth, conidiation, stress response and pathogenicity [18]. In *Aspergillus nidulans*, family 2 glycosyltransferase *CpsA*, which encodes a putative polysaccharide synthase, affects the expression of numerous genes associated with developmental processes. Δ*CpsA* shows serious defects in hyphal growth, normal sexual development and asexual development. In addition, CpsA is associated with the biosynthesis of several secondary metabolites including sterigmatocystin and penicillin [19]. However, the function of CpsA homologues has not been investigated in the *Fusarium* species. In this study, FvCpsA, which encoded a putative family 2 glycosyltransferase, was identified in *F. verticillioides*. Our results confirmed that FvCpsA played a vital role in FB1 biosynthesis, *FUM* genes expression, fungal growth, stress response and virulence in *F. verticillioides*. These findings provide a clear indicator for the exploration of glycosylation modification in the regulation of FB1 production in *F. verticillioides*, which is conducive to establishing efficient control strategies for FB1 management.

## 2. Results

### 2.1. Identification of FvCpsA in F. verticillioides

Using *A. nidulans* CpsA protein sequences as queries, homology searches in the *F. verticillioides* genome sequence database (http://fungi.ensembl.org/ *Fusarium_verticillioides*/Info/Index accessed on 1 June 2021) led to the recognition of a predicted gene, FVEG_00488, designated as *FvCPSA* in this study. The amino acid sequences of FvCpsA showed 56% identity with CpsA of *A. nidulans* (Figure S1a). To identify the homologous genes in other fungi, the *FvCpsA* amino acid sequence was used for a blastp search. Multiple sequence alignment and phylogenetic tree analyses further confirmed that CpsA homologous proteins were highly conserved in these fungal species (Figure S1b, left panel). Additionally, domain analysis showed that the homologous proteins of CpsA harbored a typical family 2 glycosyltransferase motif that was relevant for enzymatic activity [20,21] (Figure S1b, right panel). These results strongly suggest that FvCpsA is a conserved eukaryotic glycosyltransferase protein.

### 2.2. Transcription Pattern of FvCpsA in F. verticillioides

**Figure 1:**
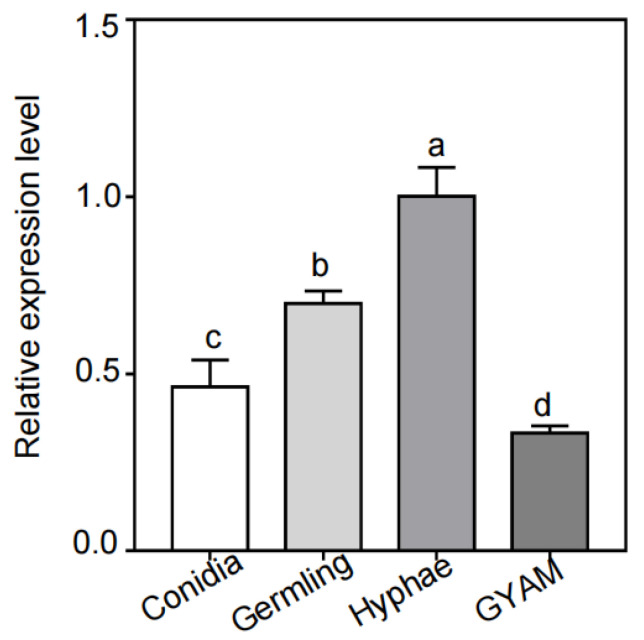
Transcriptional levels of the *FvCPSA* gene in all tested conditions, after culturing in conidia, germling, hyphae (YEPD) medium and GYAM, respectively. Expressional levels of the *FvCPSA* gene were detected by RT-PCR. The letters a, b, c and d listed in the bars show significant differences (*p* < 0.05), *n* = 3.

To obtain a detailed functional analysis of FvCpsA, first, we detected the transcription levels of the *FvCPSA* gene by quantitative reverse transcription-polymerase chain reaction (qRT-PCR) in all four different test conditions, including on conidiation (CMC), germlings (germinated spores), hyphae (yeast extract peptone dextrose-YEPD) and GYAM, a liquid medium that induces fumonisin production. As shown in Figure 1, the expression levels of the *FvCPSA* gene were significantly increased in hyphal growth conditions (YEPD). However, the expression levels of the *FvCPSA* gene showed as significantly down-regulated in the GYAM medium. These results indicated that FvCpsA might play a crucial role in the regulation of hyphal growth and FB1 production in *F. verticillioides*.

### 2.3. ΔFvcpsA Display Stunted Aerial Hyphae and Reduced Growth 

Previous studies have reported that *CPSA* homologous gene deletion mutants exhibit serious phenotype defects in *A. nidulans* and *M. oryzea* [18,19]. To identiy the function of FvCpsA in *F. verticillioides*, we generated Δ*FvcpsA* by PEG-mediated protoplast transformation method, the PCR products were amplified with the specific primer pairs (Table S1). The Δ*FvcpsA* was screened by hygromycin as the selective stress, identified with polymerase chain reaction (PCR) analysis, and was further confirmed by Southern blot analysis (Figure S2). To confirm that the defects of Δ*FvcpsA* are caused by deletion of the *FvCPSA* gene, complementation strain Δ*FvcpsA*-C was obtained by transforming the *FvCPSA* full-length fragments into Δ*FvcpsA*. 

**Figure 2:**
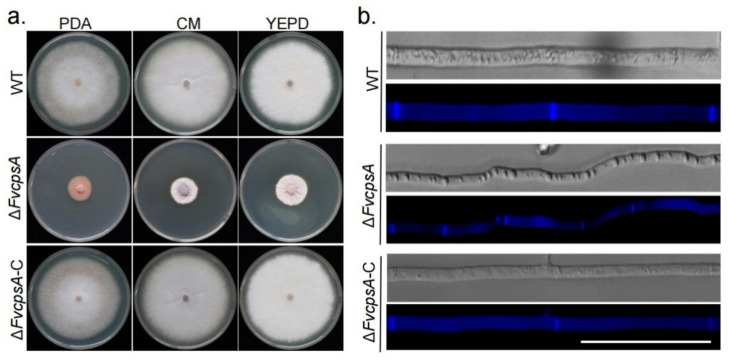
Δ*FvcpsA* is required for vegetative growth in *F. verticillioides*. (**a**) Colony morphology of the wild type, Δ*FvcpsA* and Δ*FvcpsA*-C grown on PDA (Potato Dextrose Agar), YEPD (Yeast Extract Peptone Dextrose Medium) and CM (Complete Medium) at 25 ℃ for 7 days. (**b**) The representative hyphae morphology of wild type, Δ*FvcpsA*, and Δ*FvcpsA*-C strains. Hyphae was stained by calcofluor white and photographed with a fluorescence microscope. Bar = 50 µm.

To test the function of FvCpsA for growth, Δ*FvcpsA* strain was grown on potato dextrose agar (PDA), complete medium (CM) and YEPD plates at 25 °C for 7 days. The results showed that Δ*FvcpsA* had a significantly decreased growth rate and severely stunted aerial hyphae (Figure 2a). To further examine hyphae morphology, hyphae were stained by calcofluor white (CFW) and photographed with a fluorescence microscope. It can be observed that hyphae of Δ*FvcpsA* were thinner. Meanwhile, the septa number of Δ*FvcpsA* was significantly increased compared with the wild type (WT) (Figure 2b). Phenotypic defects of the deletion mutant were also completely rescued in the Δ*FvcpsA*-C strain. Thus, these results show that FvCpsA plays an essential role in supporting the hyphal growth of *F. verticillioides*.

### 2.4. FvCpsA Is Involved in Stress Response

**Figure 3:**
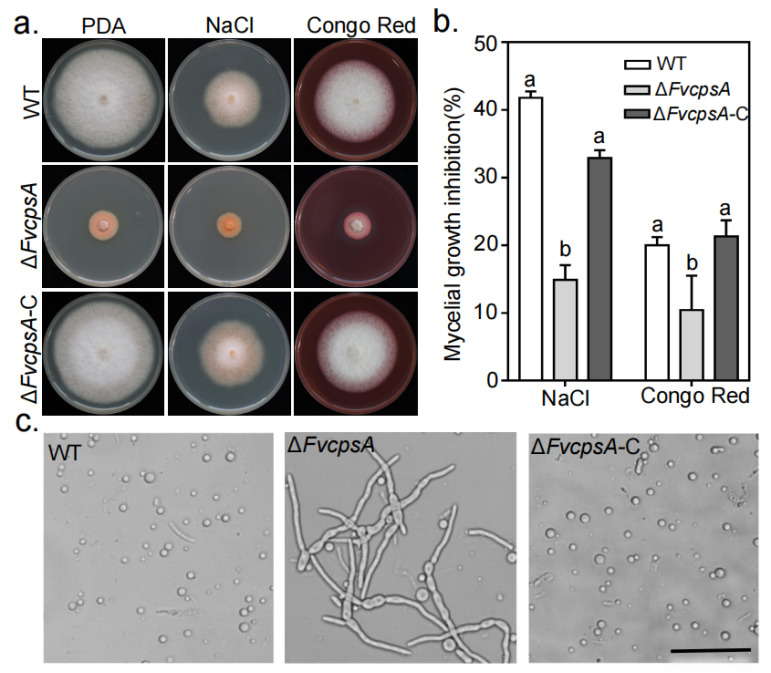
FvCpsA is involved in stress response. (**a**) Colony morphology of the wild type, Δ*FvcpsA* and Δ*FvcpsA*-C were grown on PDA agar amended with 0.7 M NaCl and 0.02% Congo Red at 25 °C for 7 days. (**b**) The inhibition rate of the mycelial growth was calculated after each tested strain was grown for 7 days under different stress conditions. The letters a, b and c listed in the bars represent significant differences (*p* < 0.05), *n* = 3. (**c**) After treatment with cellulase, lysozyme and snailase for 2 h at 28 °C, mycelia of the wild type and Δ*FvcpsA*-C, but not Δ*FvcpsA*, were well digested and produced abundant protoplasts. Bar = 50 μm.

To characterize whether FvCpsA is involved in stress responses in *F. verticillioides*, we determined the sensitivities of the Δ*FvcpsA* to environmental stress, including osmotic stress and cell wall stress generated by 0.7 M NaCl and 0.02% Congo Red, respectively. As showed in Figure 3a,b, Δ*FvcpsA* enhanced tolerance to 0.7 M NaCl and 0.02% Congo Red compared with the wild type and the Δ*FvcpsA*-C. As we can see that Δ*FvcpsA* displayed as more resistant to cell wall stress, we theorized that Δ*FvcpsA* might cause the cell wall to thicken. To test this hypothesis, the hyphae of the wild type, Δ*FvcpsA* and Δ*FvcpsA*-C were harvested with cell wall-degrading enzymes. As shown in Figure 3c, the hyphae of Δ*FvcpsA* were resistant to digestion and produced few protoplasts at 28 °C after 2 h treatment, while the hyphae of the wild type and Δ*FvcpsA*-C were well digested and generated plentiful protoplasts under the same condition. This is consistent with the results of a recent study showing that Δ*CpsA* increased tolerance to the lysing enzymes in *A. nidulans* [22]. These results suggest that FvCpsA plays a critical function in response to environmental stresses in *F. verticillioides*.

### 2.5. FvCpsA Negatively Regulates FB1 Biosynthesis in F. verticillioides

**Figure 4:**
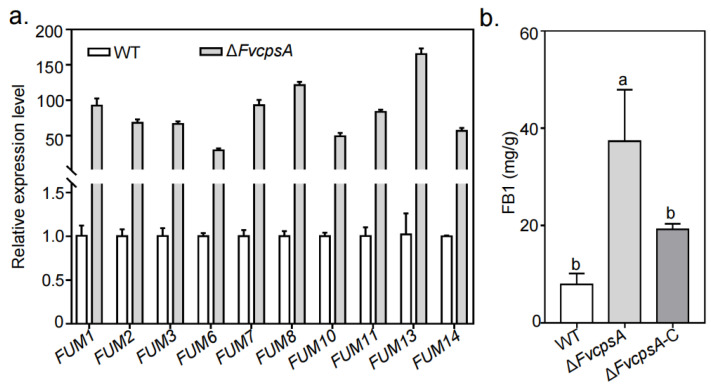
FvCpsA negatively regulates FB1 synthesis. (**a**) Relative mRNA expression levels of *FUM* genes in the tested strains. After being grown in GYAM for 3 days, the mycelia of each tested strain were collected for mRNA extraction. The β-tubulin was used as a control gene. (**b**) The contents of FB1 generated by each strain after inoculation 10 days in maize kernels. FB1 and ergosterol were quantified by HPLC. Ergosterol amount was used to normalize FB1 production. The letters a and b listed in the bars represent significant differences (*p* < 0.05), *n* = 3.

To verify the role of FvCpsA for FB1 biosynthesis in *F. verticillioides*, the transcriptional levels of *FUM* genes were detected by qRT-PCR after inoculation into the GYAM liquid medium. All selected *FUM* genes were up-regulated in the Δ*FvcpsA*. Compared with those in the wild type, the expression levels of *FUM1*, *FUM8* and *FUM13* were significantly increased in Δ*FvcpsA* (Figure 4a). Next, we assayed FB1 production by HPLC, the results showed that Δ*FvcpsA* produced more FB1 than the wild type strain (Figure 4b). Taken together, FvCpsA is negatively regulated with *FUM* genes expression and FB1 production.

### 2.6. ΔFvcpsA Displays Week Pathogenicity

**Figure 5:**
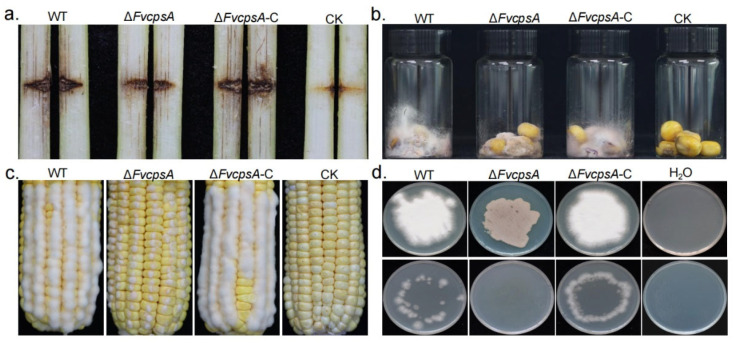
FvCpsA is essential for full virulence in *F. verticillioides*. (**a**) Maize stalk infection detection. Maize stalks were inoculated with conidial suspensions of the wild type, Δ*FvcpsA* and Δ*FvcpsA*-C. Longitudinal dissections of tested maize stalks were recorded after inoculation of 14 days. (**b**) Surface-sterilized B73 kernels were injected with conidial suspensions of the wild type, Δ*FvcpsA* and Δ*FvcpsA*-C for 10 days. (**c**) The wounded maize ear was inoculated with the same set of strains. Typical symptoms were photographed after 5 days. Sterile water served as a control. (**d**) All strains were cultured on the surface of cellophane membranes placed on the PDA medium at 25 °C for 3 days (before). After 3 days, the cellophane membranes were removed, and the plates were cultured for an additional 2 days to observe the presence of hyphal growth on the medium (after).

To determine the virulence of Δ*FvcpsA*, 8-week-old B73 maize were used for stalk rot analyses. We injected a 10-μL conidial suspension of the wild type, Δ*FvcpsA* and Δ*FvcpsA*-C into maize stalks. Stalk rot symptoms were photographed after 2 weeks inoculation. The wild type and Δ*FvcpsA*-C caused larger diseased regions and more serious deterioration of the stalk. Under the same conditions, the stalk rot symptoms of Δ*FvcpsA* were obviously reduced compared with the wild type and Δ*FvcpsA*-C (Figure 5a). The stalk rot area in Figure 5a from all replications was quantified by ImageJ software (Figure S3). To further confirm the role of FvCpsA on colonization, broken maize kernels were inoculated with a 400-μL conidial suspension of each strain. Moreover, we also carried out corn ear infection analysis. Our results showed that Δ*FvcpsA* rarely formed aerial hyphae on corn kernels and ears. Typical ear rot symptoms of Δ*FvcpsA* were not observed (Figure 5b,c). To test whether attenuated virulence is related to a penetration defect of the deletion mutant, a cellophane penetration experiment was conducted. As shown in Figure 5d, Δ*FvcpsA* did not penetrate the cellophane sheet. These results suggest that the FvCpsA plays an important role in the for colonization and virulence of *F. verticillioides*.

### 2.7. Conserved DXD and QXXRW Motifs Are Important for FvCpsA Function

**Figure 6:**
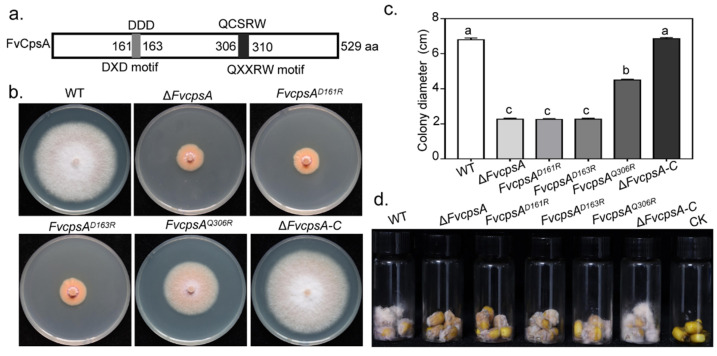
The DXD and QXXRW motifs are required for hyphal growth and pathogenicity of *F. verticillioides*. (**a**) Schematic drawing of the FvCpsA protein with the conserved DXD and QXXRW motifs. The two conserved motifs are shown in grey and black. (**b**) Functional analysis of the DXD and QXXRW motifs. Colony morphology of the wild type, Δ*FvcpsA*, *FvcpsA^D161R^*, *FvcpsA^D163R^*, *FvcpsA^Q301R^* and Δ*FvcpsA*-C were grown on PDA agar at 25 °C for 7 days. (**c**) Bar chart indicates the colony diameters of the strains cultured on the PDA medium for 7 days. The letters a, b and c listed in the bars represent significant differences (*p* < 0.05), *n* = 3. (**d**) Assessment of pathogenicity of the *FvcpsA^D161R^*, *FvcpsA^D163R^* and *FvcpsA^Q301R^* mutants on surface-sterilized B73 kernels. Photos were taken 10 days post-inoculation.

It has been reported that family 2 glycosyltransferase (GT2) harbored the conserved DXD and QXXRW motifs in *Bacillus subtilis*. Recently, by means of constructing point strains pGT2^D156R^, pGT2^D158R^ and pGT2^Q301R^, DXD and QXXRW motifs were demonstrated to be essential for MoGT2 function in *M. oryzae* [18]. Sequence analysis indicated that FvCpsA also contained the conserved DXD and QXXRW motifs (Figure 6a). To verify the function of these motifs in FvCpsA, we constructed site-directed mutagenesis strains, *FvcpsA^D161R^*, *FvcpsA^D163R^* and *FvcpsA^Q301R^*. The molecular identification of transformants was performed by PCR (Figure S4a). Semi-quantitative RT-PCR was employed to detect the expression of the *FvCPSA* gene in point mutation strains (Figure S4b). By observing the phenotype of the point mutant strains, we found that *FvcpsA^D161R^* and *FvcpsA^D163R^* failed to recover the growth defects of Δ*FvcpsA*, indicating the DXD motif was important for FvCpsA function in regulating vegetative growth. In addition, we further confirmed that the introduction of FvcpsA^D163R^ fragment also could partially complement the vegetative growth defect of Δ*FvcpsA* (Figure 6b,c). Next, we assayed the pathogenicity of the point mutation strains by inoculating their conidia onto damaged maize kernels, with the wild type conidia as a control. The results demonstrated that *FvcpsA^D161R^*, *FvcpsA^D163R^* and *FvcpsA^Q301R^* had weakened pathogenicity on corn kernels compared with the wild type, especially *FvcpsA^D161R^* and *FvcpsA^D163R^* (Figure 6d). The above results indicate that the DXD motif is essential and the QXXRW motif is partially essential for FvCpsA function in regulating the growth and pathogenicity of *F. verticillioides*.

## 3. Discussion

In this study, family 2 glycosyltransferase FvCpsA was identified in *F. verticillioides*. CpsA homologues are widely distributed among several filamentous fungi, however, they are absent from organisms that cannot form true aerial hyphae. Δ*FvcpsA* presented a great difference in growth phenotype compared with the wild type. Targeted deletion of *FvCPSA* resulted in serious defects in vegetative growth, environmental stress tolerance, invasive growths and pathogenicity. As a family 2 glycosyltransferase protein, FvCpsA harbors the conserved DXD and QXXRW motifs. Site-directed mutagenesis analysis confirms that these motifs are required for the full function of FvCpsA. The above results are consistent with other fungal pathogens that the *CpsA* homologous genes are functionally characterized [17,18,19,23,24,25].

Hyphal growth is very important for the pathogenicity of plant pathogenic fungi. Prior to responding to plant immunity, most plant-pathogenic fungi need first adhere to the host surface and then grow hyphae through host tissues and cells to cause diseases [26]. It has been reported that family 2 glycosyltransferase, GT2, is necessary for hyphal growth in *Z. tritici* and *F. graminearum* [17]. In *M. oryzae*, Δ*MoGT2* reduced mycelial growth by shortening the distance between two septa of vegetative hyphae [18]. In *F. verticillioides*, the Δ*FvcpsA* strain showed significantly reduced hyphal growth on YEPD, CM and PDA plates. The results of the CFW staining assay showed Δ*FvcpsA* also reduced interseptal distances, confirming FvCpsA also plays a critical role in fungal hyphae development. Δ*FvcpsA* showed greatly reduced colonizing capability on corn stalks, kernels and ears in comparison with the wild type and the Δ*FvcpsA*-C strain. Through cellophane penetration experiments, it was found that Δ*FvcpsA* lost its invasive growth ability. In *F. graminearum*, the deletion of the *FgMAP1* gene resulted in the Δ*FgMAP1* strain losing invasive growth ability and having weakened pathogenicity [27]. As is shown in Figure 5d, Δ*FvcpsA* was not capable of penetrating the cellophane sheet. It is speculated that the impaired ability of invasive growth is the cause of the weakened virulence of Δ*FvcpsA*. Additionally, the hyphal growth defect of the mutant may partially influence the virulence of *F. verticillioides* in planta. Taken together, our results further illustrate that FvCpsA plays a key role in regulating the pathogenicity of *F. verticillioides*.

A cell wall is required to maintain cell shape and is critical for cell expansion [28]. In *M. oryzae* and *A. nidulans*, the deletion of the *CPSA* homologous gene caused increases in sensitivity to different stresses, even changing the cell wall ultrastructure [18,19]. In *Z. tritici*, loss of *ZtGT2* altered outer cell wall structure, but displayed no altered sensitivity to stress response [17]. However, in this study, the lack of FvCpsA increased resistance to 0.7 M NaCl and 0.02% Congo Red in *F. verticillioides*. Δ*FvcpsA* decreased the sensitivity to cell wall-degrading enzymes, indicating that the lack of FvCpsA may affect the structure of the cell wall of *F. verticillioides*.

During fungal development, *F. verticillioides* produces mycotoxin FB1. It has been reported that deletion of some genes, including *FvSEC4* and *FvDIM5*, led to the production of more FB1 [9,29]. In this study, Δ*FvcpsA* was found to increase FB1 production compared with that of the wild type and the Δ*FvcpsA*-C strain. Furthermore, transcriptional levels of 10 *FUM* genes were significantly increased in Δ*FvcpsA*. Consistent with the above FB1 production and expression of *FUM* genes, the *FvCPSA* gene showed the lowest expression in inducing FB1 production conditions (GYAM), implicating FvCpsA negatively regulates FB1 biosynthesis in *F. verticillioides*. Since the synthesis of FB1 is strongly regulated at the transcriptional level, it is speculated that the absence of FvCpsA may up-regulate the expression of *FUM* genes, which in turn increases the production of FB1. Previous studies have revealed that CpsA can influence secondary metabolism production by affecting the expression of upstream regulators *veA*, *laeA* and *afI*R which is necessary for the activation of other genes in the secondary metabolism gene cluster in *A. nidulans* [19].

## 4. Conclusions

In summary, we identified a family 2 glycosyltransferase FvCpsA in *F. verticillioides*. The data indicates that FvCpsA is involved in regulating hyphal growth, environmental stress tolerance and pathogenicity processes. More importantly, FvCpsA has been confirmed to negatively regulate FB1 biosynthesis. Future investigation should analyze the molecular mechanism of FvCpsA negatively regulated FB1 synthesis.

## 5. Materials and Methods

### 5.1. Fungal Strains and Culture Conditions

The wild type strain *F. verticillioides* was used as an experimental strain. All transformants constructed in this study were grown on a PDA medium at 25 °C [30].

### 5.2. Constructed Gene Deletion and Complemented Strains

The split-marker approach was performed to generate gene deletion mutants. Primers listed in Table S1 were used to amplify the fragments of the hygromycin B phosphotransferase gene, as well as upstream and downstream sequences of the *FvCPSA* gene. These products were used to transform into the wild type as previous described protocols [30]. Δ*FvcpsA* was identified by PCR and a Southern blot assay (Figure S2).

For complementation assays, the full-length *FvCPSA* gene was amplified by specific primers (Table S1). The PCR products were cloned into pGTN plasmid with the One Step cloning kit. The complementation plasmid was transformed into protoplasts of Δ*FvcpsA* to generate the complementation strain Δ*FvcpsA*-C.

To construct the *FvcpsA^D161R^* mutant, the upstream and downstream of site-directed mutants were amplified from wild type genomic DNA with primer pairs, respectively (Table S1). G418 fragment was amplified with primer pair Trpc-neo-F/R from PYF11 plasmid. Primer pairs listed in Table S1 were used to amplify the fusion fragments, and the PCR products were transformed into the Δ*FvcpsA* protoplast to generate the *FvcpsA^D161R^* mutant. Similar approaches were used to construct other site-directed mutants.

### 5.3. Statistical Analysis Method

Data analysis was performed by SPSS Statistics 21.0 software. One-way ANOVA and Fisher’s LSD test was used for statistical analysis (*p* < 0.05). All the charts in the article were made by GraphPad Prism 7.0.

### 5.4. CFW Staining Assays

All tested strains were cultured on the PDA solid medium. The sterilized coverslip was inserted into the mediums at an angle of 45°. The coverslip was removed when the hypha covered it. Calcofluor white (1 g/L) and 10% NaOH were added to the coverslip for 1 min. Hyphae morphology was photographed using a LEICA DM6B microscope.

### 5.5. qRT-PCR Assays

To detect the transcriptional levels of *FvCPSA* in different conditions, the spores of wild type were inoculated into a 100 mL CMC liquid medium at 25 °C (180 rpm) for 3 days. The medium was then filtered into a clean 50 mL centrifuge tube by using a sterile funnel with two-layer Miracloth and centrifuged at 5000 rpm for 15 min to remove the supernatant and collect spores. A portion of the collected spores were added to 100 mL YEPD liquid medium and incubated at 25 °C (180 rpm) for 12 h and filtered to collect fresh germling. Hyphae of wild type were cultured in 100 mL YEPD liquid medium at 25 °C (180 rpm) for 3 days, then collected. Hyphae of wild type were harvested after 3 days of incubation in 100 mL liquid GYAM medium. RNA was extracted by TRIzol reagent for all the samples collected above.

To detect *FUM* genes expression, fresh mycelia of wild type and Δ*FvcpsA* were cultured in a 100 mL GYAM medium for 3 days. Subsequently, mycelia were collected by filtration with a Miracloth, RNA was isolated with the TRIzol reagent from mycelia of each sample. For qRT-PCR analysis, the cDNA was synthesized with the TransScript One-Step gDNA Removal and cDNA Synthesis SuperMix kit, following the instructions provided by the manufacturer. Relative transcriptional levels of genes were calibrated by the 2^-ΔΔCT^ approach and the β-tubulin gene of *F. verticillioides* served as a control [2].

### 5.6. Fumonisin B1 Production Assays

For FB1 production assay, corn kernels of similar size and shape were placed in 50 mL falcon tubes and sterilized by a protocol previously described [31]. Sterilized kernels, which were wounded on the endosperm area, were inoculated with the conidial suspension of each tested strain (400 μL, 10^6^ spores/mL) and cultured at 12-h-light/12-h-dark for 10 days at 28 °C. Samples were then extracted with 10 mL of acetonitrile/water (50/50, *v/v*) overnight. To determine the content of ergosterol, add 10 mL of chloroform: methanol (2:1, *v/v*) in each vial, vortex samples well for 2 min and then incubate for 24 h. The supernatant was collected and filtered with a 0.45 μm nylon membrane. FB1 and ergosterol were measured by an HPLC system [31]. The amounts of FB1 were normalized by ergosterol contents. These experiments were carried out with three biological replicates.

### 5.7. Pathogenicity Assays

To examine the virulence of each strain on maize ears, conidial suspension (10^6^ conidia/mL) was injected into maize ears with sterile toothpicks. The control maize ears were inoculated with sterilized water. The maize ears were incubated at 12-h-light/12-h-dark under 100% humidity for 5 days at 25 °C [32]. The maize kernels pathogenicity assays referred to fumonisin B1 production assays. To test the virulence of each strain on maize stalks, stalks grown with 8-week-old B73 maize were wounded by a sterile toothpick and inoculated with 10 µL conidial suspension (10^6^ conidia/mL). The control was injected with sterilized water. Each strain included six replicates. After inoculation for two weeks, stalks were split longitudinally to examine the rot extent of maize [9,33].

### 5.8. Penetration of Cellophane Sheets Assays

100 µL conidial suspension (10^6^ conidia/mL) was grown for 3 days at 25 °C on plates that with a layer of cellophane membrane on the medium surface and then removed. The cellophane and plates were cultured for an additional 2 days to observe whether invasive hyphae appeared on the PDA medium.

## Data Availability

The data presented in this study are available in this article.

## Supplementary Materials

The following are available online at https://www.mdpi.com/article/10.3390/toxins13100718/s1. Figure S1. Identification of the family 2 glycosyltransferase proteins in *F. verticillioides*. (a) Amino acid alignment of FvCpsA and its ortholog CpsA in *A. nidulans*. (b) Phylogenetic relationship and domain analysis of family 2 glycosyltransferase. Phylogenetic tree was constructed by the neighbour-joining method with Mega 7 software based on the amino acid sequences from fungal species including *F. oxysporum*, *F. proliferatum*, *F. graminearum*, *C. gloeosporioides*, *M. oryzae*, *N. crassa*, *A. nidulans*, and *Z. tritici*. Conserved domains are indicated. Figure S2. Construction and verification of Δ*FvcpsA*. (a) PCR identification of the Δ*FvcpsA*. (b) Southern blot analysis of the Δ*FvcpsA*. The upstream fragment of *FvCPSA* was used as the probe. Δ*FvcpsA* had an anticipated 4210 bp band, while 5808 bp band was presented in the wild type. Figure S3. Statistical analysis of lesion area of longitudinally dissected maize stalk in Figure 5a. The letters a, b and c listed in the bars represent significant differences (*p* < 0.05), *n* = 6. Figure S4. Identification of point mutant strains (a) PCR identification of the point mutants. (b) Semi-quantitative analysis of *Fv**CPSA* expression. β-tubulin was served as the control. Table S1. The primers listed in this study.
