# Supplementary material for: Glycosyltransferase FvCpsA Regulates Fumonisin Biosynthesis and Virulence in Fusarium verticillioides
Source: Toxins (Basel). 2021 Oct 11;13(10):718. doi: 10.3390/toxins13100718 (PMC8539240; doi:10.3390/toxins13100718)
Supplement: Supplementary file 1 [file toxins-13-00718-s001.zip › toxins-1376674-supplementary.pdf]

Supplementary Materials: Glycosyltransferase FvCpsA Regulates Fumonisin Biosynthesis and Virulence in *Fusarium verticillioides*

Qi Deng[1], Hanxiang Wu[1], Qin Gu[2], Guangfei Tang[1] * and Wende Liu[1] *

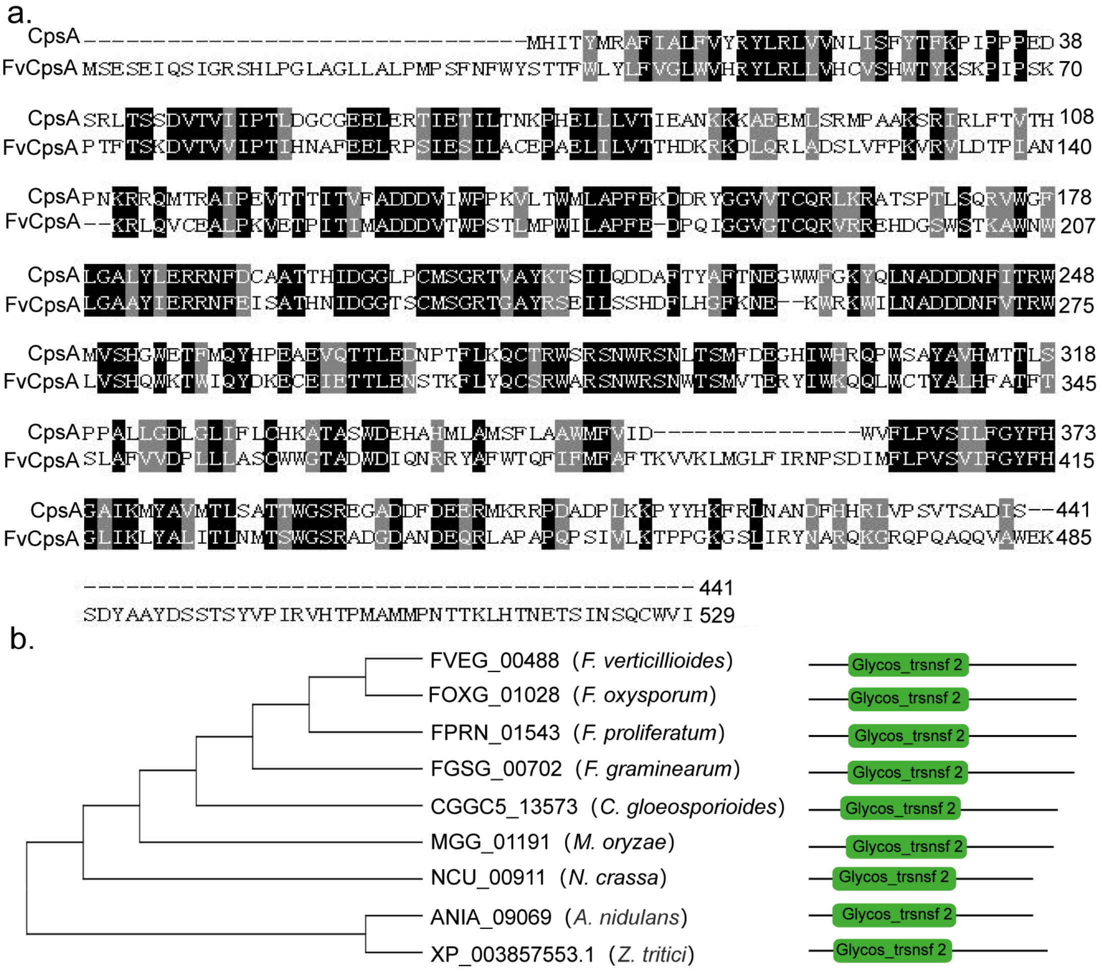

Figure S1. Identification of the family 2 glycosyltransferase proteins in *F. verticillioides*. (a) Amino acid alignment of FvCpsA and its ortholog CpsA in *A. nidulans*. (b) Phylogenetic relationship and domain analysis of family 2 glycosyltransferase. Phylogenetic tree was constructed by the neighbour-joining method with Mega 7 software based on the amino acid sequences from fungal species including *F. oxysporum*, *F. proliferatum*, *F. graminearum*, *C. gloeosporioides*, *M. oryzae*, *N. crassa*, *A. nidulans*, and *Z. tritici*. Conserved domains are indicated.

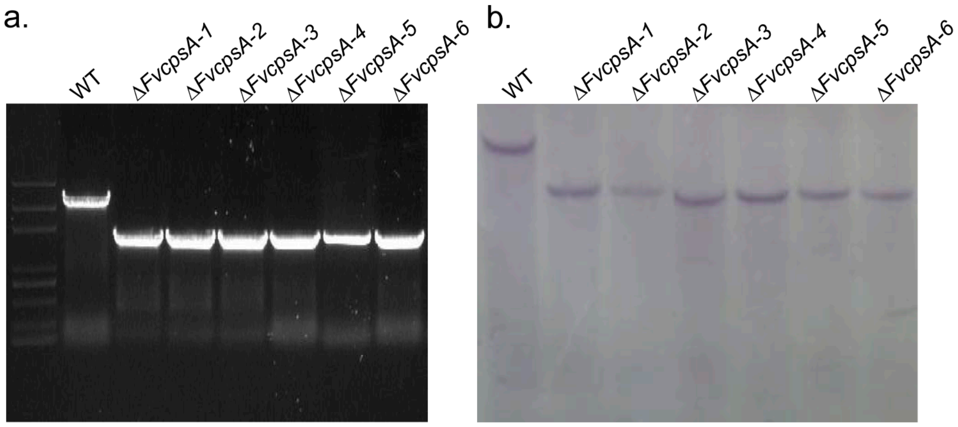

Figure S2. Construction and verification of Δ*FvcpsA*. (a) PCR identification of the Δ*FvcpsA*. (b) Southern blot analysis of the Δ*FvcpsA*. The upstream fragment of *FvCPSA* was used as the probe. Δ*FvcpsA* had an anticipated 4210 bp band, while 5808 bp band was presented in the wild type.

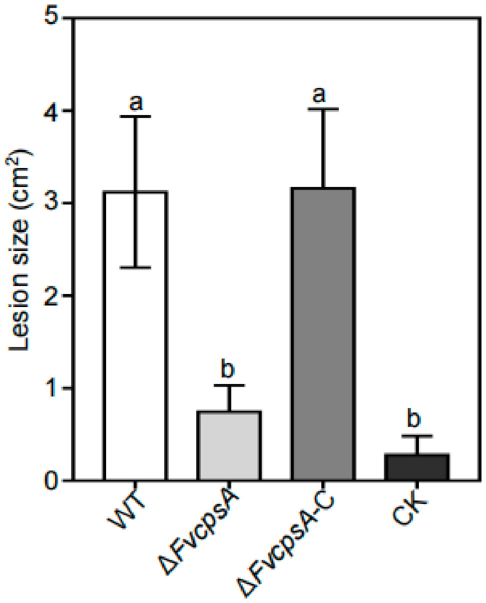

Figure S3. Statistical analysis of lesion area of longitudinally dissected maize stalk in Figure 5a. The letters a, b and c listed in the bars represent significant differences ($P<0.05$), N=6.

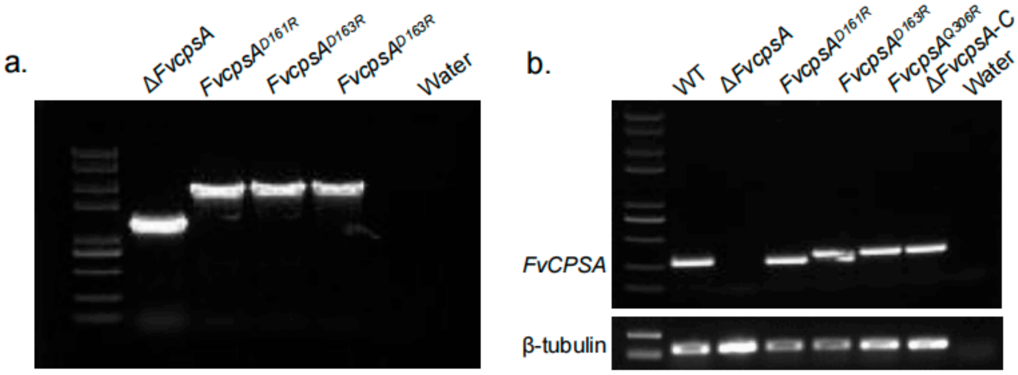

Figure S4. Identification of point mutant strains (a) PCR identification of the point mutants. (b) Semi-quantitative analysis of *FvCPSA* expression. β-tubulin was served as the control.

Table S1. A list of primers used in this study.

Code	Name	Sequence (5'to 3')	Products
P1	HYG-F	CTTGGCTGGAGCTAGTGGAGGT	A pair of PCR primers to amplify full Hygromycin B phosphotransferase gene fragments
P2	HYG-R	CCCGGTCGGCATCTACTCTATTC	
P3	FvcpsA-LBCK	CCAGACAGGGACCTCGGATTT	Upstream fragment of *FvcpsA*
P4	FvcpsA-LB-R	ACCTCCACTAGCTCCAGCCAAGTAGTCGTGGTGGCACTGGTAGG	
P5	FvcpsA-RB-F	GAATAGAGTAGATGCCGACCGGGGGGCGCAGACATTTCATACC	Downstream fragment of *FvcpsA*
P6	FvcpsA-RBCK	CCAGGGACCTGCATCCAATT	
P7	FvcpsA-LB-F	CGCTCAGTGGCCTTTGGTT	Partial hygromycin B phosphotransferase gene fuse with left flanking regions of *FvcpsA*
P8	HYG-R$_1$	GGATGCCTCCGCTCGAAGTA	
P9	HYG-F$_1$	CGTTGCAAGACCTGCCTGAA	Partial hygromycin B phosphotransferase gene fuse with right flanking regions of *FvcpsA*
P10	FvcpsA-RB-R	GCCCGCAGAAAGCTAGACA	
P11	FvcpsA -ID-F	CTCAAATCATCCAACCCCTCG	Identification of *FvcpsA* deletion transformants
P12	FvcpsA -ID-R	ACTCTGTGTGTGGGTGGTGCTGT	
P13	FV t-F	TGCGATGCTGAATGGCTGTC	A pair of PCR primers to amplify probe fragments used for Southern blot
P14	FV t-R	TAGTCGTGGTGGCACTGGTAGG	
P15	FvcpsA -com-F	GGGAACAAAAGCTGGGTACCCTCCAAGTCAGGTTGTGCT	A pair of PCR primers to amplify fragment used for construction of the *FvcpsA* completment, vector under its own promoter
P16	FvcpsA-com-R	GTAACGTTAAGTGCGGCCGCCCAACGATGCCATTTTAGG	
P17	UP-F1	CGCTCAGTGGCCTTTGGTT	Upstream fragment of DXD/QXXRW motif
P18	RXD-R1	AGGTCACATCATCGCGCGCCAT	
P19	DXR-R1	CCAGGTCACGCGATCGTCCGCC	
P20	RXXRW-R1	AACGAGAGCAGCGATACAAAA	
P21	RXD-F2	ATGGCGCGCGATGATGTGACCT	Downstream fragment of DXD/QXXRW motif
P22	DXR-F2	GGCGGACGATCGCGTGACCTGG	
P23	RXXRW-F2	TTTTGTATCGCTGCTCTCGTT	

P24	up-R2	GCATTGATGTGTTGACCTCCGTTAT ACCCGCCTCAGAAGT	
P25	Trpc-neo-F	GGAGGTCAACACATCAATGCT	A pair of PCR primers to amplify full G418 fragments
P26	Trpc-neo-R	TCAGAAGAACTCGTCAAGAAG	
P27	Down-F3	TTCTTGACGAGTTCTTCTGA GGGCGCAGACATTTCATACC	Downstream fragment of *FvcpsA*
P28	Down-R3	GGCCCGCAGAAAGCTAGACA	
P29	UP-F4	ATGTTGCAGATTGATGAGTCC	A pair of PCR primers to fuse all fragments of point mutantion
P30	Down-R4	CCGCGTCAGAATCCATATCAT	
P31	ID-pm-R	CGAGTGTAACACGTCAAGCCCTAA T	Identification of point mutantion transformants
P32	ID-pm-F	GCAATGAACCAGAGCCAAAGC	
P33	β-tubulin-F	GCTCTTCCGTCCCGACAACTT	qRT-PCR primers of *ACTIN*
P34	β-tubulin-R	CAATCGCAGCCCTCAGCCTCA	
P35	FvcpsA-F	ATTAGCACTGCCAATGCCTTCTTTC	qRT-PCR primers of *FvcpsA*
P36	FvcpsA-R	CCTTACTCGTGAATGTCGGTTTGCT	
P37	FUM1-F	TGCTGCCCTGTATCACAACCA	qRT-PCR primers of *FUM1*
P38	FUM1-R	AATGTGCGCTTGATCCAGTT	
P39	FUM2-F	AAGTGCTCGGGGAGCGGGTT	qRT-PCR primers of *FUM2*
P40	FUM2-R	TCGGGGCATAACTCTATATCG	
P41	FUM3-F	ACTGATTTCACCGAGGCCAA	qRT-PCR primers of *FUM3*
P42	FUM3-R	AGCGGACCGGAAGCTTCT	
P43	FUM6-F	TCTCTTGTTCTTTGGCTGTCG	qRT-PCR primers of *FUM6*
P44	FUM6-R	TCAATTTCTAGCAGCATCGG	
P45	FUM7-F	GCATGGAGAGACAAGTTGCA	qRT-PCR primers of *FUM7*
P46	FUM7-R	TCTGATGAAACTGGGCTTCGT	
P47	FUM8-F	GCGCTTGAGAGACGACTGGCC	qRT-PCR primers of *FUM8*
P48	FUM8-R	GGTTGGCGCATGCACTGAGC	
P49	FUM10-F	TTTGGAACCCAATGGCGAT	qRT-PCR primers of *FUM10*
P50	FUM10-R	TTTCGGCAGGGCTGATTTTT	
P51	FUM11-F	AAGGGGGGAAGATAGGCACT	qRT-PCR primers of *FUM11*
P52	FUM11-R	ATTACGAGTCTTAGCGAGCG	
P53	FUM13-F	AAACCATGGGATGGTATCAGG	qRT-PCR primers of *FUM13*
P54	FUM13-R	TTTCTGCTGAGCCGACATCAT	
P55	FUM14-F	AAGAGGTGCTAAAGACAGCCA	qRT-PCR primers of *FUM14*
P56	FUM14-R	ACTCAGGAGCTGCGACTGATA	
